# HPV infection and *p53* inactivation in pterygium

**Published:** 2009-06-01

**Authors:** Yi-Yu Tsai, Chi-Chung Chang, Chun-Chi Chiang, Kun-Tu Yeh, Pei-Liang Chen, Chi-Huang Chang, Ming-Chih Chou, Huei Lee, Ya-Wen Cheng

**Affiliations:** 1 Institute of Medicine, Chung Shan Medical University, Taichung, Taiwan; 2 Department of Ophthalmology, China Medical University Hospital, Taichung, Taiwan; 3 Department of Pathology, Changhua Christian Hospital, Changhua, Taiwan; 4 Department of Pharmacy, Tung’s Taichung Memorial Hospital, Taichung, Taiwan; 5 Institute of Medical & Molecular Toxicology, Chung Shan Medical University, Taichung, Taiwan

## Abstract

**Purpose:**

Our recent report indicated that tumor suppressor gene (*p53*) mutations and protein aberrant expression were detected in pterygium. Inactivation of *p53* by Human papillomavirus (HPV) 16/18 E6 plays a crucial role in cervical tumorigenesis. In this study, we further speculate that *p53* inactivation may be linked with HPV infection in pterygium pathogenesis. To investigate the involvement of HPV 16/18 E6 in *p53* inactivation in pterygium, the association between HPV 16 or HPV 18 infection, the HPV E6 oncoprotein, and p53 protein expression was analyzed in this study.

**Methods:**

HPV 16/18 infection was detected by nested-polymerase chain reaction (nested-PCR), the *p53* mutation was detected by direct sequencing, and the p53 and the HPV 16/18 E6 proteins were studied using immunohistochemistry on 129 pterygial specimens and 20 normal conjunctivas.

**Results:**

The HPV 16/18 was detected in 24% of the pterygium tissues (31 of 129) but not in the normal conjunctiva, and the HPV16/18 E6 oncoprotein was detected in 48.3% of HPV 16/18 DNA-positive pterygium tissues (15 of 31). In addition, p53 protein negative expression in pterygium was correlated with HPV16/18 E6 oncoprotein expression but not with a *p53* mutation.

**Conclusions:**

HPV 16/18 E6 contributes to HPV-mediated pterygium pathogenesis as it is partly involved in *p53* inactivation and is expressed in HPV DNA-positive pterygium.

## Introduction

Pterygium has long been considered to be a degenerative disease. However, following the finding that the p53 protein is abnormally expressed in the epithelium, pterygium is now thought to be an ultraviolet (UV)-related, uncontrolled cell proliferation that is similar to a tumor [1-7].

The integration of high-risk Human papillomavirus 16/18 (*HPV 16/18*) DNA into the host chromosome to express the E6 protein plays a crucial role in HPV-induced cervical carcinogenesis [8-10]. E6 has many functions that may contribute to its oncogenic potential. The classical function of E6, which is relevant to cellular immortalization, is binding to the tumor suppressor p53, thereby inducing p53 degradation [11]. The role of p53 is to safeguard the integrity of the genome by inducing cell cycle arrest or apoptosis upon DNA damage [12]. As a transcription factor, p53 upregulates target genes involved in coordinating these responses such as *p21^WAF1/CIP1^*, a cyclin-dependent kinase (CDK) inhibitor that acts on cyclin E/cdk2 complexes, and murine double minute (*mdm2*) [13,14]. Therefore, p53 inactivation by E6 leads to chromosomal instability and increases the probability of an HPV-infected cell evolving toward malignancy [11].

Several reports revealed that low or no frequency of HPV infection was found in pterygium [15-24] while some data from Italy, Greece, the United Kingdom, and Brazil showed that significantly higher frequencies of HPV infection (24%–58.3%) were determined in pterygium than in conjunctivas [18,20,24]. The association between HPV infection and pterygium was then reasonably suggested to be geography- and race-dependent. In our preliminary studies, the low frequencies of *p53* mutation and p53 positive immunostaining were detected in pterygium [25]. This phenomenon is quite similar to the pattern of *p53* alteration found in cervical cancer [26]. Therefore, we hypothesize that high-risk HPV 16/18 infection may be involved in pterygium pathogenesis in Taiwan.

In this report, we analyzed p53 protein expression and gene mutation in pterygium in comparison with HPV 16/18 infection and E6 oncoprotein expression in pterygium tissues to understand whether the HPV infection was involved in pterygium pathogenesis.

## Methods

### Patients and controls

Pterygial samples were harvested from 129 patients who were undergoing pterygium surgery and submitted a written informed consent approved by the Institutional Review Board. Patients in whom the apex of the pterygium had invaded the cornea by more than 1 mm were included in this study. Normal conjunctival samples were collected from the superior conjunctivas of 10 patients without pterygium and the medial conjunctivas of another 10 patients without pterygium and pinguecula, all of them were undergoing cataract or vitreoretinal surgery, and these normal samples were included as controls. All specimens were fixed in formalin and were paraffin-embedded.

### Nested polymerase chain reaction

Genomic DNA was prepared from a tissue section and isolated by conventional phenol-chloroform extraction, ethanol precipitation, and finally dissolved in 20 μl of sterile distilled water. HPV viral DNA was first amplified with type consensus primers, MY09 and MY11 [27], followed by a second round of amplification with type specific primers flanking the L1 region to identify the subtype (sequences of MY09: 5′-GCM CAG GGW CAT AAY AAT GG-3′ and MY11: CGT CCM ARR GGA WAC TGA TC; Type 16 primers: 5′-TAC TAA CTT TAA GGA GTA CC-3′ and 5′-GTG TAT GTT TTT GAC AAG CAA TT-3′; sequences of Type 18 primers: 5′-CCA AAT TTA AGC AGT ATA GC-3′ and 5′-TTG TAC AAA ACG ATA TGT ATC CA-3'). The final polymerase chain reaction (PCR) product (10 μl) was loaded onto a 2% agarose gel, stained with ethidium bromide, and visualized under UV illumination. Appropriate negative and positive controls were included in each PCR reaction. A part of *β-actin* in all samples was amplified to exclude false-negative results while DNA preparations from SiHa cells (containing HPV 16) and HeLa cells (containing HPV 18) were used as positive controls.

### Immunohistochemistry

All sections were deparaffinized in xylene, sequentially rehydrated in alcohol, and washed in phosphate-buffered saline. Sections used for p53 and HPV 16/18 E6 detection were heated in a microwave oven twice for 5 min in citrate buffer (pH 6.0). Mouse anti-p53 monoclonal antibody (at a dilution of 1:200; DAKO, Copenhagen, Denmark) and goat anti-HPV 16/18 E6 antibody (at a dilution of 1:50; Santa Cruz Biotechnology, Santa Cruz, CA) were used as the primary antibodies. The detail protocols were described in our previous reports [28-30]. Negative controls that did not include the primary antibodies were set up. The cutoff value for immunohistochemical analysis was set at 10%, which means the positive cutoff is when more than 10% of the cells are stained. The results were evaluated independently by three observers and scored for the percentage of positive or negative.

### *p53* mutation analysis

Mutations in exons 4, 5, 6, 7, and 8 of *p53* were determined by direct sequencing, and the methods were described in our previous report [25]. DNA was extracted from the paraffin-embedded pterygium tissues by traditional proteinase K digestion and phenol-chloroform extraction. Target sequences were amplified in a 50 μl reaction mixture containing 20 pmol of each specific primer, 2.5 units of Taq polymerase (Takara Shuzo, Shiga, Japan), 0.5 mM dNTPs, 5 μl of PCR reaction buffer, and 1 μl of genomic DNA as the template. *β-actin* was used as an internal control. An initial cycle was performed for 5 min at 94 °C, and then 35 cycles each for 40 s at 94 °C, 40 s at 54 °C, and 1 min at 72 °C. PCR products were then purified with the QIAEX Gel Extraction Kit (Qiagen, Germantown, MD). Approximately 20 ng of purified PCR product (QIAquick PCR Purification kit; Qiagen) was directly sequenced using the Big Dye Terminator Reaction kit, version 3.1 (Applied Biosystems, Foster City, CA) and sequenced by an auto-sequencing system (Applied Biosystems 3100 Avant Genetic Analyzer; Applied Biosystems). All of the *p53* mutations were confirmed by direct sequencing of both strands.

### Statistical analysis

Statistical analysis was performed using the SPSS statistical software program (SPSS Inc., Chicago, IL). Fisher’s exact test was applied for statistical analysis. A p value less than 0.05 was considered to be statistically significant.

## Results

There were 76 males and 53 females in the pterygium group (age range=50–83 years, mean=64.7 years), and 10 males and 10 females in the control group (age range=55–75 years, mean=67.2 years).

### HPV 16/18 DNA detected in pterygium but not in control group

To elucidate the association between HPV infection and pterygium development, nested PCR with two different sets of primers was used to examine the existence of HPV 16/18 DNA in pterygium tissues and the control group. As shown in Table 1, the detection frequency of HPV 16/18 DNA in pterygium (31 of 129, 24%) was higher than that of control patients (0 of 20, 0%; p=0.014). When the study subjects were stratified by gender and age, HPV 16/18 DNA detection frequency was not different. The higher prevalence of HPV 16/18 in pterygium suggests that HPV infection may play a role in pterygium pathogenesis.

**Table 1:** The differences of HPV 16/18 infection between pterygium tissues and conjunctiva control.

**Parameters**	**Patients (%)**	**Control (%)**	**p value**
HPV 16/18 DNA			
Negative	98 (76.0%)	20 (100%)	
Positive	31 (24.0%)	0 (0%)	0.014

HPV 16/18 DNA was detected by nested-PCR.

### HPV 16/18 E6 protein were detected in HPV-positive pterygium tissues

**Figure 1:**
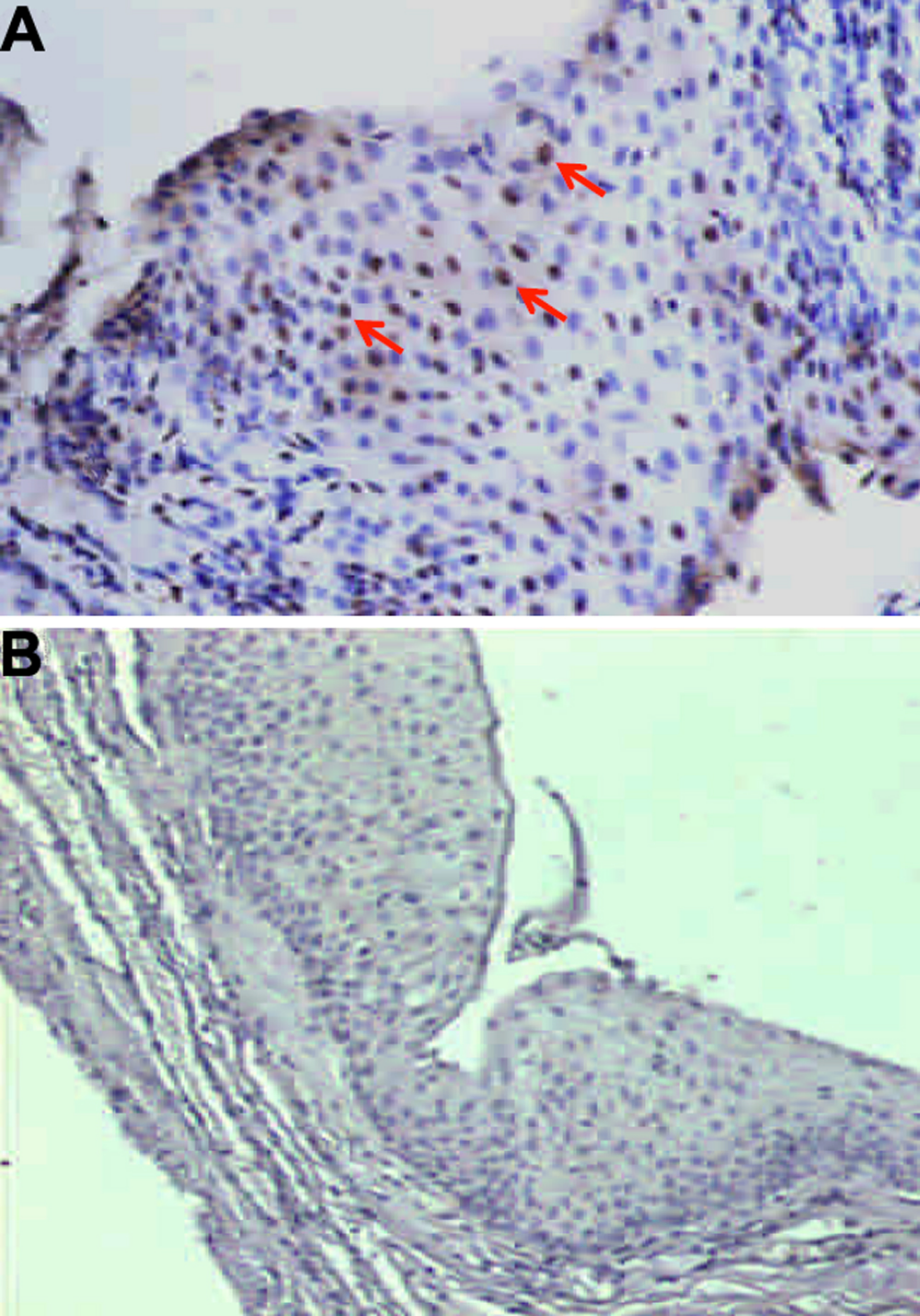
Representative positive and negative immunostaining for HPV 16/18 E6 protein in paraffin sections of pterygium. Representative positive HPV 16/18 E6 immunostaining is shown in (**A**). The brown color pointed out by the red arrow indicates a HPV 16/18 positive signal. Negative HPV 16/18 E6 immunostaining is shown in (**B**).

After successfully detecting HPV infection in pterygium tissues, we attempted to determine by immunohistochemistry whether HPV 16/18 E6 is expressed to verify the association between HPV infection and HPV16/18 E6 expression. HPV 16/18 E6 protein staining was limited to the nuclei of the epithelial layer (Figure 1). Our data showed that HPV 16/18 E6 was only detected in 48.3% (15 of 31) of HPV 16/18 DNA positive pterygium tissues. Not any HPV-negative pterygium and control group had HPV 16/18 E6 protein expression (Table 2; p<0.0001).

**Table 2:** Relationship of HPV 16/18 infection and E6 oncoprotein expression in pterygia tissues.

**HPV 16/18 DNA**	**HPV 16/18 E6 oncoprotein**		**p value**
	**Negative**	**Positive**	
**Negative**	98	0	
**Positive**	16	15	<0.0001

### p53 inactivation associated with HPV 16/18 E6 protein expression

**Figure 2:**
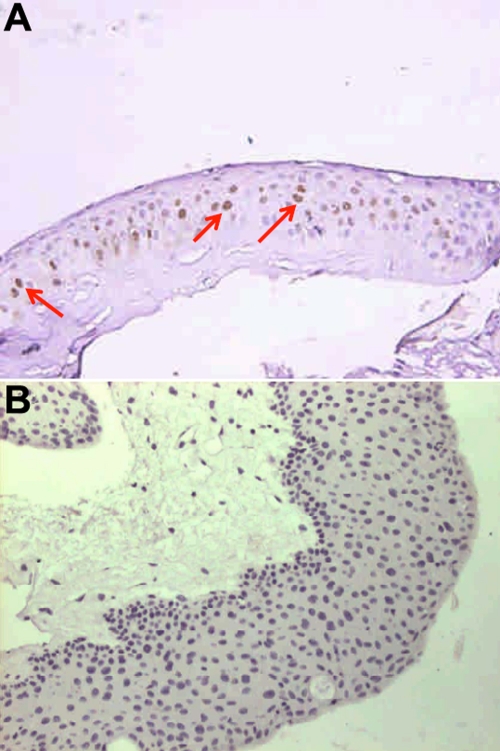
Representative positive and negative immunostainings for p53 protein in paraffin sections of pterygium. Representative positive p53 immunostaining is shown in (**A**), negative p53 immunostaining is shown in (**B**). The brown color pointed out by red arrow indicates p53 positive signal.

Immunohistochemistry was used to detect the HPV 16/18 E6 oncoprotein and p53 expressions (Figure 2) in the 129 pterygium tissues and to elucidate whether HPV 16/18 E6 affects p53 protein expression. In addition, *p53* mutations were also analyzed by direct sequencing to verify the correlation with p53 protein expression. As shown in Table 3, the frequency of p53 negative expression was higher than positive expression (12 of 15, 80.0% versus 3 of 15, 20.0%, respectively) in 15 HPV 16/18 E6 positive patients. Of these three HPV+/p53+ patients, two had a *p53* mutation. The site of the *p53* mutation for patient 5 was at exon 4, codon 107, and for patient 6, it was at exon 7, codon 234. Additionally, *p53* mutations were detected in 9.3% (12 of 129) of the pterygium group but not detected in the control group. No correlation was observed between p53 protein expression and its gene mutation. This result was concordant with our previous report [25].

**Table 3:** Relationship of HPV 16/18 E6 oncoprotein, *p53* mutation, and p53 protein expression in pterygium tissues.

**Patient number**	**HPV E6 protein**	**p53 protein**	***p53* mutation**
1	+	-	Wild type
2	+	-	Wild type
3	+	-	Wild type
4	+	-	Wild type
5	+	+	C→G (exon 4, codon 107)
6	+	+	T→A (exon 7, codon 234)
7	+	-	Wild type
8	+	-	Wild type
9	+	-	Wild type
10	+	-	Wild type
11	+	-	Wild type
12	+	-	Wild type
13	+	-	Wild type
14	+	+	Wild type
15	+	-	Wild type

The p53 and HPV E6 proteins were detected by immunohistochemistry. The cutoff value of immunohistochemical analysis was set at 10%, which means that those with more than 10% of their cells stained are considered to be positive. The *p53* mutation was detected by direct DNA sequence.

## Discussion

High-risk HPV E6 has many functions that may contribute to its oncogenic potential. The classical function of E6, which is relevant to cellular immortalization, is the binding to the tumor suppressor, p53, thereby inducing p53 degradation [11].The integration of high-risk HPV16/18 DNA into the host chromosome to express the E6 protein plays a crucial role in HPV-induced cervical carcinogenesis [8-10]. Throughout literature, the prevalence of HPV infection in pterygia varies from 0%−54% [15-24]. All of these reports detected HPV infection at a DNA level but not the HPV E6 oncoprotein. As far as we know, this is the first study to report that the HPV 16/18 E6 oncoprotein was detected in pterygia tissues. In this study, the HPV 16/18 E6 protein was detected in 11.6% (15 of 129) of the pterygium patients, but the HPV 16/18 DNA detection rate was 24.0% (31 of 129). E6 oncoprotein expression was detected in only 48.4% of the HPV-positive pterygia cells. In previous studies, high-risk HPV E6 gene expression has been evaluated by reverse transcription polymerase chain reaction (RT–PCR), RNA in situ hybridization, and immunohistochemistry [31-37]. Although RT–PCR is more sensitive in the detection of HPV E6 than immunohistochemistry, results from the RT–PCR detection of HPV E6 may be misleading due to contamination of other cells. Therefore, in this study, we used the immunohistochemistry data to provide direct evidence showing that *p53* inactivation in pterygium may be correlated with HPV 16/18 E6 oncoprotein expression (Table 3). Additionally, our data show that p53 expression was not associated with a *p53* mutation (Table 3), which is inconsistent with a previous study [38] showing that positive *p53* expression was due to the increased protein stability by *p53* missense mutations. In addition, E6 variants with mutations in the NH_2_-terminal region lost the ability to bind with E6-associated protein (E6AP) and failed to cause p53 protein degradation [39]. Therefore, the patients with both HPV 16/18 E6 and p53 protein positive expressions may correlate with also having a *p53* mutation or the E6 variant. Based on these results, we hypothesize that inactivation of *p53* by a high-risk HPV E6 oncoprotein may play a role in a HPV-mediated pterygium pathogenesis.

In conclusion, our study demonstrates for the first time that high-risk HPV E6 oncoprotein is indeed expressed in pterygium and is linked to p53 protein negative expression. Moreover, p53 negative expressions were not correlated with gene mutations. These data provide molecular evidence that HPV 16/18 E6 contributes to HPV-mediated pterygium pathogenesis as it is partly involved in *p53* inactivation and is expressed in HPV DNA-positive pterygium.
